# Combining visual analytics and case-based reasoning for rupture risk assessment of intracranial aneurysms

**DOI:** 10.1007/s11548-020-02217-9

**Published:** 2020-07-04

**Authors:** Lena Spitz, Uli Niemann, Oliver Beuing, Belal Neyazi, I. Erol Sandalcioglu, Bernhard Preim, Sylvia Saalfeld

**Affiliations:** 1 Faculty of Computer Science, Otto-von-Guericke University Magdeburg, Universitätsplatz 2, D-39106 Magdeburg, Germany; 2 University Hospital Magdeburg, Germany, Leipziger Str. 44, D-39120 Magdeburg, Germany; 3 Forschungscampus STIMULATE, Magdeburg, Germany

**Keywords:** Visual analytics, Case-based reasoning, Intracranial aneurysms, Rupture risk assessment

## Abstract

**Purpose:**

Medical case-based reasoning solves problems by applying experience gained from the outcome of previous treatments of the same kind. Particularly for complex treatment decisions, for example, incidentally found intracranial aneurysms (IAs), it can support the medical expert. IAs bear the risk of rupture and may lead to subarachnoidal hemorrhages. Treatment needs to be considered carefully, since it may entail unnecessary complications for IAs with low rupture risk. With a rupture risk prediction based on previous cases, the treatment decision can be supported.

**Methods:**

We present an interactive visual exploration tool for the case-based reasoning of IAs. In presence of a new aneurysm of interest, our application provides visual analytics techniques to identify the most similar cases with respect to morphology. The clinical expert can obtain the treatment, including the treatment outcome, for these cases and transfer it to the aneurysm of interest. Our application comprises a heatmap visualization, an adapted scatterplot matrix and fully or partially directed graphs with a circle- or force-directed layout to guide the interactive selection process. To fit the demands of clinical applications, we further integrated an interactive identification of outlier cases as well as an interactive attribute selection for the similarity calculation. A questionnaire evaluation with six trained physicians was used.

**Result:**

Our application allows for case-based reasoning of IAs based on a reference data set. Three classifiers summarize the rupture state of the most similar cases. Medical experts positively evaluated the application.

**Conclusion:**

Our case-based reasoning application combined with visual analytic techniques allows for representation of similar IAs to support the clinician. The graphical representation was rated very useful and provides visual information of the similarity of the *k* most similar cases.

## Introduction

Case-based reasoning (CBR) solves problems by applying experience gained from previous cases. It enables a computer to imitate a human expert and can adapt the treatment of past cases to a newly presented one. In clinical research and practice, CBR finds its use in many different areas, such as diagnosis, classification and therapy planning. It has been employed for diagnosis and treatment planning of hypertension, to manage and treat diabetic patients, to diagnose heart failure or coronary heart diseases, as well as for the interpretation of biomedical images [5, 8, 13, 18].

**Fig. 1:**
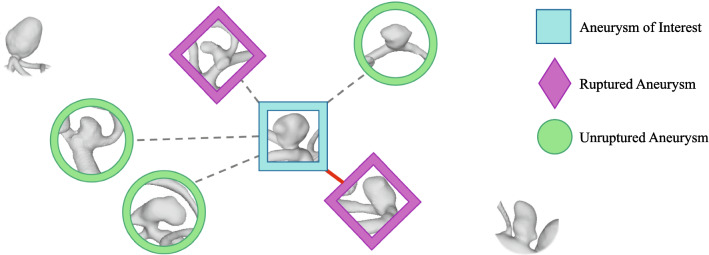
Illustration of the classification problem. The similarity between different intracrancial aneurysms is mapped to Euclidean distances of the morphological parameter feature space; aneurysms next to each other are more similar than distant aneurysms. The aneurysm of interest is depicted in the center, and its *k* nearest neighbors (*k* = 5) including their rupture states are highlighted. The most similar aneurysm is a ruptured one, connected with a red line, whereas the remaining ones in the *k* neighborhood are connected with dashed lines. Note that of the five aneurysms only two are ruptured and three are unruptured, but that the ruptured ones have a smaller distance and, thus, are more similar.

We developed an interactive visual exploration tool to facilitate CBR for the treatment of intracranial aneurysms (IAs). These pathologic dilatations of the intracranial vessel wall have an inherent rupture risk and may cause subarachnoidal hemorrhages. However, the go-to treatments like endovascular treatment or microsurgical clipping need to be considered carefully, as they may entail unnecessary treatment complications for aneurysms with low rupture risk. Thus, accurate rupture risk prediction is crucial for an optimal treatment strategy and currently an active research area [4, 10].

Particularly due to the strong variations of patient-specific anatomy, the medical expert relies on previous experiences rather than pre-defined categorization schemes. Hence, the motivation of using CBR for IA treatment covers two aspects. First, we aim to determine the rupture risk. Second, we aim to detect similar IAs with respect to their geometrical shape to support the clinical expert’s treatment planning.

Our main focus is the visual presentation of the similarity of the previous cases, as illustrated in Fig. 1. We do not only show the *k* most similar cases, but rather provide their similarity within graphical representations and suggest a classification. To account for strong differences, regarding aneurysms at different localizations in the brain, we only compare aneurysms at the same localization. On the one hand, aneurysm localization is crucial for treatment planning, i.e., for determination of the access path. On the other hand, aneurysm rupture risk varies for different localizations [10, 14], and the treatment strategy depends on the individual rupture risk as well. Furthermore, outlier identification and elimination as well as feature selection is integrated in the application.

In summary, our application identifies the *k* *most similar* IAs to the reference case, i.e., the new aneurysm of interest comprising the visual analytics techniques:

- a heatmap-based overview of the database to estimate the overall agreement as well as possible outliers in the database,
- a circle- and force-directed graph visualization to show the *k* most similar aneurysms alongside their similarity,
- a scatterplot-based visualization incorporating feature–feature correlation and feature–outcome correlation to further steer the selection of similarity criteria,
- and a rupture risk prediction based on the *k* most similar cases and three classifier variants.

We conducted a structured evaluation with six medical experts and provide their feedback and suggestions.

## Related work

Case-based reasoning has found its uses in many areas, from bankruptcy prediction over marketing to risk analysis and fault prediction, as well as in the health sector [6, 8, 13]. Several CBR systems are already in use, be it for consultation, diagnosis or planning in healthcare [8, 13, 18]. For example, Benamina et al. [3] improved the classification of diabetic patients with their CBR system that includes a fuzzy decision tree for cases retrieval. In the context of intracranial aneurysms, CBR has been used for their detection in MR angiography [15], and for their rupture risk evaluation in the context of a statistical model [9, 11].

A general model of CBR has been presented by Aamodt et al. [1] comprising four key steps: retrieve, reuse, revise and retain. First, a problem is presented as a new case, which is then compared to previous cases, one or more of which are then retrieved. The previous cases are then reused or adapted to the new case to present a solution. The solution is applied to the real world or gets reviewed by an expert and is revised based on its success or failure. The experience gained from this process is then retained by adding the case to the database.

Chuang et al. [8] used CBR as well as various data mining methods, such as back-propagation neural networks, classification and regression trees, or logistic regression, to diagnose liver diseases that are difficult to detect. For CBR, each case was defined as a feature vector, which could be weighted and normalized to calculate the Euclidean distance between cases to find the most similar ones. A classification as healthy or diseased was made by calculating the overall similarity to all diseased and all healthy cases and comparing the two. This CBR approach was then combined with the data mining methods which increased sensitivity and specificity compared to the performance of each method on its own. Our application was inspired by the presented approach, but we were aiming at CBR for IA treatment and rupture risk prediction.

When analyzing the IA rupture risk, many studies evaluated their morphology with respect to the rupture state [7, 12, 16]. Niemann et al. [17] investigated the potential of 22 morphological features for aneurysm rupture risk prediction. Although the best model had an accuracy of only 69%, several features showed high association toward rupture risk.

Recently, Detmer et al. [10] introduced an aneurysm rupture probability model based on patient characteristics, i.e., age and gender, aneurysm location, morphology and hemodynamics. A study by Ishibashi et al. [14] further cemented the influence of an aneurysm’s location and size on the rupture risk, as well as a patient’s medical history. In addition, the Multiple Aneurysms AnaTomy CHallenge 2018 (MATCH) was conducted to provide an overview of state-of-the-art blood flow simulations as well as rupture risk prediction [4]. As a result, the simulation setups of the participating groups revealed very similar boundary conditions of the simulations, but clear differences were reported regarding morphological and hemodynamical parameters of the aneurysms.

In contrast to these approaches, we focus on the concept of similarity, highlighting the individual aneurysm cases, and illustrate their similarities in graph layouts.

## Medical image datasets

Our database covers approximately 200 aneurysms from patients that underwent digital subtraction angiography, acquired in the daily clinical practice. In order to compare only aneurysms at the same localization, together with medical cooperation partners 17 possible localizations were classified, see Table 1. In addition, we integrated 24 cases from the Aneurisk repository [2] yielding a total of 51 aneurysms at the anterior communicating artery which were used for this study. However, our tool can be used for other databases with arbitrary case number as well.

**Table 1:** Localization classes for intracranial aneurysms

Class	Detail
*Location of aneurysms*	
1	A1
2	Acom
3	M1
4	M2
5	MCA Bif
6	pericall A
7	Pcom
8	BAS tip
9	PICA
10	ICA
11	Carotid T
12	PCA
13	AchoA
14	paraophth.
15	intraophth A
16	callosomarg A
17	other

A1—A1 segment of anterior cerebral artery, Acom—anterior communicating artery, M1—M1 segment of middle cerebral artery (MCA), M2—M2 segment of MCA, MCA Bif—MCA bifurcation, pericall A—pericallosal artery, Pcom—posterior communicating artery, BAS tip—tip of basilar artery, PICA—posterior inferior cerebellar artery, ICA—internal carotid artery, Carotid T—terminus of carotid artery, PCA—posterior cerebral artery, AchoA—anterior choroidal artery, paraophth A—paraophthalmic artery, infraophth A—intraophthalmic artery, callosomarg A—callosomarginal artery

Our approach can deal with arbitrary quantitative features. For the proposed method, we focused on morphological parameters since they have been established for rupture risk prediction [12]. We applied a semiautomatic neck curve extraction to obtain the morphological parameters [19]. Based on previous work [17], we decided to employ the following parameters: *A*—surface area of the aneurysm sac, *V*—volume of the aneurysm sac, $OA_1$—area of the ostium, $OA_2$—area of the ostium projected onto a plane, $D_\mathrm{max}$—maximum diameter, $H_{max}$—maximum height, $W_\mathrm{max}$—maximum width perpendicular to $H_\mathrm{max}$, $H_\mathrm{ortho}$—height perpendicular to the ostium center, $H_\mathrm{ortho2}$—same as $H_\mathrm{ortho}$, but no intersections with the aneurysm wall are allowed, $W_{ortho}$—maximum width parallel to the projected ostium plane, $N_\mathrm{max}$— maximum ostium diameter, $N_\mathrm{avg}$ average ostium diameter, $AR_1$ —aspect ratio: $H_\mathrm{ortho} / N_\mathrm{max}$ and $AR_2$ = $H_\mathrm{ortho} / N_\mathrm{avg}$, $V_\mathrm{CH}$—volume of the convex hull of the aneurysm sac, $A_\mathrm{CH}$—area of the convex hull of the aneurysm sac, *EI*— ellipticity index, *NSI*—non-sphericity index, *UI*–undulation index, $\alpha$—larger angle between centerline and dome, $\beta$ —smaller angle between centerline and dome, $\gamma$—angle at the aneurysm dome. See also the studies [17, 19] for detailed information.

## *k*-NN-based prediction of aneurysm rupture risk

**Fig. 2:**
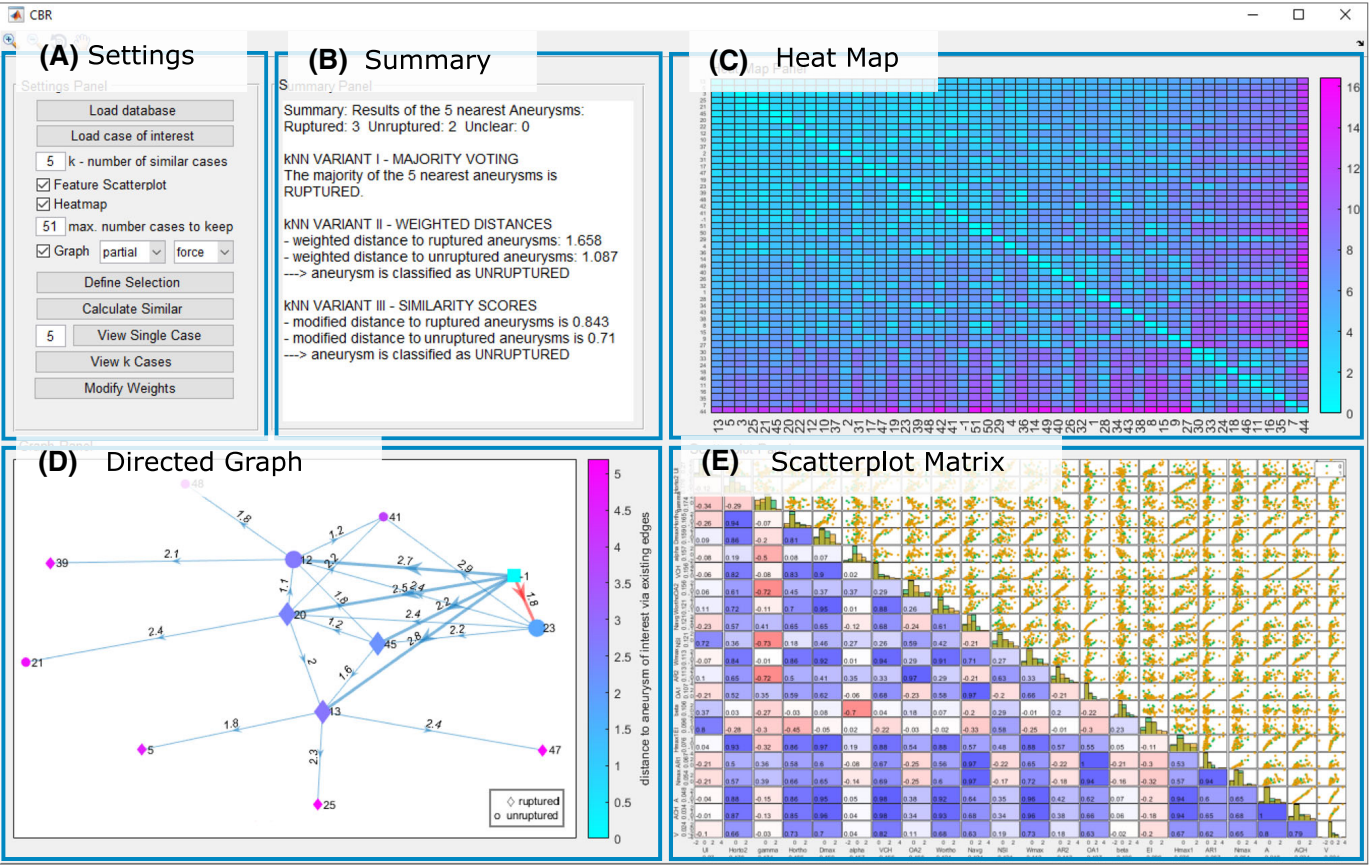
The graphical user interface. **a** Settings panel, **b** Summary panel, **c** Heatmap panel, **d** Directed Graph panel, **e** Adapted scatterplot matrix panel

We provide a computational assessment of aneurysm rupture risk based on morphological features by employing three variants of *k*-nearest neighbor-based (*k*-NN) classification. For this purpose, a new aneurysm, hereafter denoted as aneurysm of interest (AOI), is compared to cases from a reference database containing both ruptured and unruptured aneurysms at the same location. Rupture status prediction is based on the *k* most *similar* cases. The range of each feature was normalized by (z-score) standardization. The *dissimilarity* between the two aneurysms *x*, *q* is calculated as

1 $$\begin{aligned} \mathrm{dist}(x,q)=\sqrt{f_{w_l}\sum \limits _{l=1}^{N} (x_l - q_l)^2}, \end{aligned}$$

where *N* is the number of features, $x_l$ is the value of the *l*th feature of *x*, and $f_{w_l}$ is the feature’s weight. Per default, all feature weights equal 1. The user can increase or decrease a feature weight, e.g., maximum aneurysm diameter, via the settings panel (A), see Fig. 2. Afterward, the remaining feature weights are normalized such that the $\sum _{l=1}^{N} f_{w_l} = N$.

The first classifier variant is a simple *k*-nearest neighbor classifier which assigns a class label $y\in \{\text {ruptured,unruptured}\}$ to the AOI (*x*). First, the algorithm calculates the pairwise dissimilarity between *x* and all aneurysms from the database $q\in D$. Then, the set of *k* nearest aneurysms of *x*, $D_k\subseteq D$, is selected and the rupture risk classification ${\hat{y}}$ is obtained based on the majority class of *x*’s nearest neighbors:

2 $$\begin{aligned} {\hat{y}}={{\,\mathrm{argmax}\,}}_j \sum \limits _{i=1}^{k} I(y_i = j), \end{aligned}$$

where *j* is a class label, $y_i$ is the class label of the *i*th nearest neighbor, and *I* is the indicator function.

This ordinary *k*-nn classifier does not take distances into account, i.e., every nearest neighbor has the same impact on the classification, regardless of its actual distance to the AOI which might be imprecise and prone to outlier cases. Hence, our second *k*-nn variant incorporates the actual distances as weights. The predicted class label ${\hat{y}}$ is calculated as:

3 $$\begin{aligned} {\hat{y}}={{\,\mathrm{argmin}\,}}_j \sum \limits _{i=1}^{k} I(y_i = j) \cdot w_{i,j} \cdot \mathrm{dist}(x,q_i), \end{aligned}$$

where *j* is a class label, $y_i$ is the class label of the *i*th nearest neighbor, and *I* is the indicator function. The weight *w* of the *i*th nearest neighbor and class *j* is calculated as

4 $$\begin{aligned} w_{i,j}={\left\{ \begin{array}{ll} w_i=\left( \sum \limits _{o=1}^{M_j} \mathrm{dist}(x, q_o) - \mathrm{dist}(x, q_i)\right) \\ /{\sum \limits _{o=1}^{M_j} \mathrm{dist}(x, q_o)} &{} \text {if } y_i = j \\ 0 &{} \text {else} \end{array}\right. },\nonumber \\ \end{aligned}$$

where $M_j$ is the number of aneurysms of class *j* in the set of *k*-nearest neighbors of *x*. Thus, for each class, all weights sum up to 1 and each aneurysm is associated with a weight inversely proportional to its distance.

Our third *k*-NN-based classifier is adapted from Chuang et al. [8], who also used a distance-weighted method for similarity calculation for their *CBR* approach for liver disease diagnosis. Here, all distances were normalized via min–max scaling into the range [0,1]. The classification is defined as

5 $$\begin{aligned} {\hat{y}}={{\,\mathrm{argmin}\,}}_j \sum \limits _{i=1}^{k} I(y_i = j)\cdot \mathrm{dist}(x,q_i) / M_j, \end{aligned}$$

where $M_j$ is the number of aneurysms of class *j* in the set of *k*-nearest neighbors of *x*.

## Graphical user interface

In this section, we describe the components of the graphical user interface (GUI) for the identification of the most similar aneurysms for a given aneurysm of interest. The application was developed with MATLAB 2019a (MathWorks, Natick, USA) and comprises five components (depicted in Fig. 2):

(A) a settings panel, e.g., for database import and selection of number of neighbors *k*,
(B) a summary panel presenting the result of the analysis of the *k* neighbors,
(C) a heatmap visualization showing the pairwise dissimilarities of all IAs,
(D) a circle- and force-directed graph visualization to highlight the relationships among the *k* most similar aneurysms alongside their dissimilarity,
(E) a scatterplot matrix showing pairwise feature correlations and association toward rupture risk.

In the settings panel (A) on the top left, the user can load the database, load an aneurysm of interest (AOI) and set the parameter *k*, the number of nearest neighbor cases in the database to be considered. *k* must be within the range of 2 and $|D|-1$, and it is empirically set to 5 by default. Since localization of IAs plays a crucial role for treatment planning, e.g., the access path determination, we created subgroups of IAs with the same localization. Therefore, the user has to select the AOIs localization of one of the pre-defined classes (recall Table 1). In the following, 51 IAs at the anterior communicating artery are analyzed.

Subsequently, a summary panel (B) which displays the rupture risk prediction and three visualization panels are shown: a heatmap (C), a graph (D) and a scatterplot matrix view (E), recall Fig. 2. The presented application allows for an interactive *k*-NN search for CBR including an adapted feature and outlier detection which will be explained in the following.

**Fig. 3:**
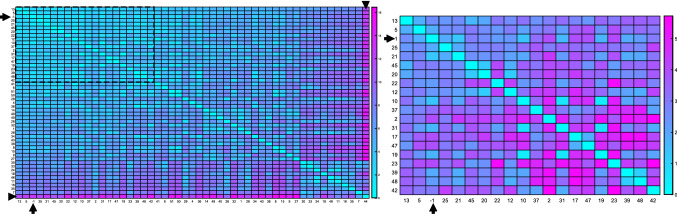
Heatmap visualizations of IAs with their pairwise color-coded Euclidean distance of the morphological data. Aneurysms are sorted based on the average sum of their pairwise similarity, i.e., aneurysms on the upper left of the heatmap are very similar to all other cases, whereas aneurysms at the bottom right are not very similar to any other cases. Left, the heatmap for all IAs is shown. The AOI is marked with arrows, and aneurysm with id 44 (arrowhead) has the lowest similarity to all other aneurysms. To account for possible outlier aneurysms, only a user-defined number of aneurysms is kept for the subsequent analyses. For 20 aneurysms (see dashed rectangle on the left), the heatmap is depicted on the right

The first visual representation is a heatmap (C), see Fig. 3, showing the pairwise color-coded distances between all IAs in the database, sorted by average distance to all other IAs. The diagonal shows the distance of an aneurysm to itself, which is zero and the lightest color in the heatmap. Hovering or clicking on the entries highlights row and column aneurysms, and a pop-up appears that shows the X and Y values, which here is the index of the aneurysms, as well as the distance. Further basic interaction is possible, like dragging single columns or rows across the map to directly compare to others, or re-sorting the entire heatmap according to one specific aneurysm. Since there are only two values that shall be represented, i.e., most to least similar, a map over two colors was chosen. A temperature scale is intuitive for this purpose, and the “cool” layout provided by MATLAB was chosen as it shows the best contrast and displays the shift from most to least similar. With this visualization technique, the user can easily identify which aneurysms are outliers, see the marked example in Fig. 3. To cope with larger database sizes, we do automatically keep only a user-defined amount of aneurysms. However, the user can still reject a single aneurysm due to low similarity or just keep all datasets for the subsequent analyses.

**Fig. 4:**
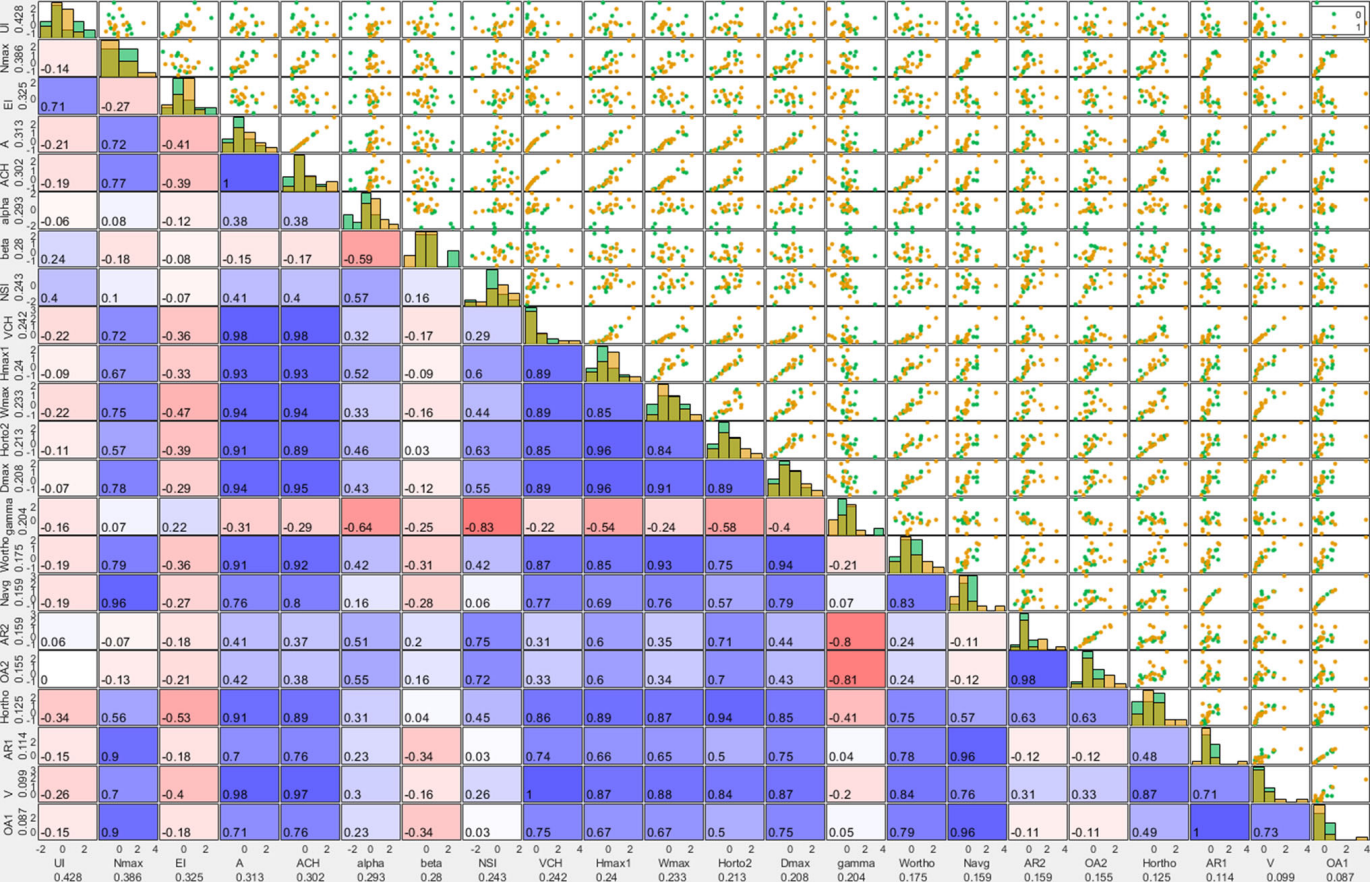
The scatterplot matrix including $\Delta _\mathrm{info}$ and PCC. Each row and each column represent a morphological feature. Labels provide the name of the feature and its $\Delta _\mathrm{info}$. The upper triangle matrix shows pairwise scatterplots, the lower triangle matrix the color-coded PCC (red = 1; white = 0; blue = -1)

The next visualization is a scatterplot matrix (E), where each row and each column represent a morphological feature (see Fig. 4). The upper triangle of the matrix shows pairwise scatterplots of the morphological features. Each point in the scatterplots represents an aneurysm color-coded by its rupture status class. The features are sorted according to their information gain $\Delta _\mathrm{info}$ toward the rupture status. Here, the information gain of feature *f* measures the decrease in impurity *H* of *D* toward the rupture status when splitting *D* into *R* partitions and is calculated as

6 $$\begin{aligned} \Delta _\mathrm{info}(f) = H(D) - \sum _{r=1}^R \frac{M_r}{M} H(D_r), \end{aligned}$$

where *D* is the dataset, *R* is the number of partitions, and $D_r$ is the *r*th partition with $\bigcup _{r=1}^R D_r = D$. The impurity *H* of a partition $D_r$ is measured using Shannon entropy as

7 $$\begin{aligned} H(D_r) = -\sum _{j} p(j|D_r)\log _2 p(j|D_r), \end{aligned}$$

where $p(j|D_r)$ is the ratio of aneurysms of class *j* in $D_r$. Each continuous feature is split into $R=2$ partitions, with $\forall q\in D_1:q_f\le \tau _f$ and $\forall q\in D_2:q_f > \tau _f$, where $\tau _f$ is the cutoff value for *f* that yields the highest $\Delta _\mathrm{info}(f)$ over all unique values of *f*.

**Fig. 5:**
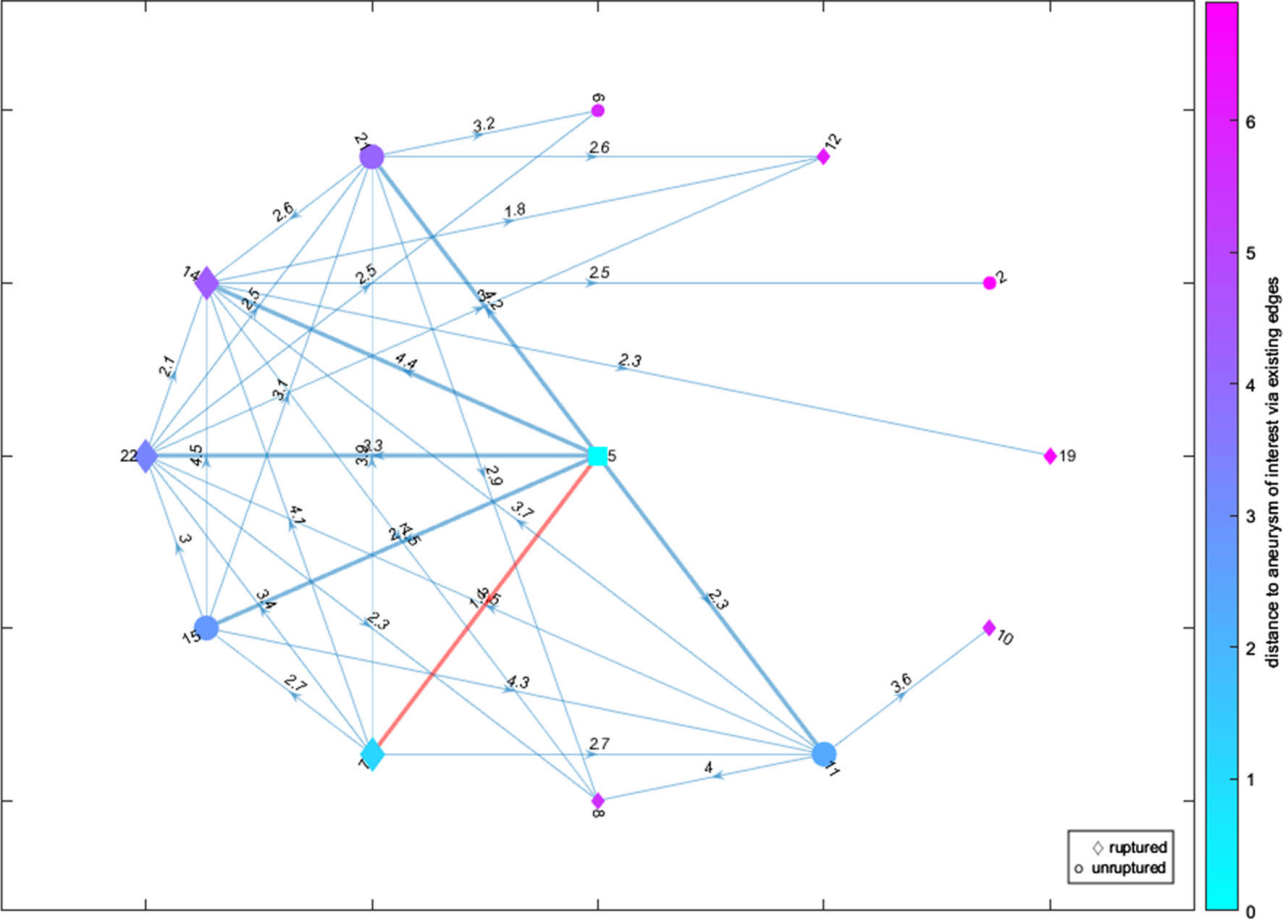
A circle graph layout representing the *k* = 6 nearest neighbors (enhanced edges) including the highlighting of the most similar aneurysm (red edge) and a color-coding of the distance based on the color assigned to circular or diamond shapes representing the rupture state. The AOI is depicted as cyan-colored square

The lower triangle of the scatterplot matrix shows the pairwise color-coded Pearson correlation coefficient (PCC) of the row and column feature. The PCC between two morphological parameters is visualized via color and saturation. Through $\Delta _{info}$, PCC and the distribution of the scatterplots, the scatterplot matrix gives the user a sense of which features might be important for the classification of the AOI, and they can chose to exclude single features for the similarity calculations.

**Fig. 6:**
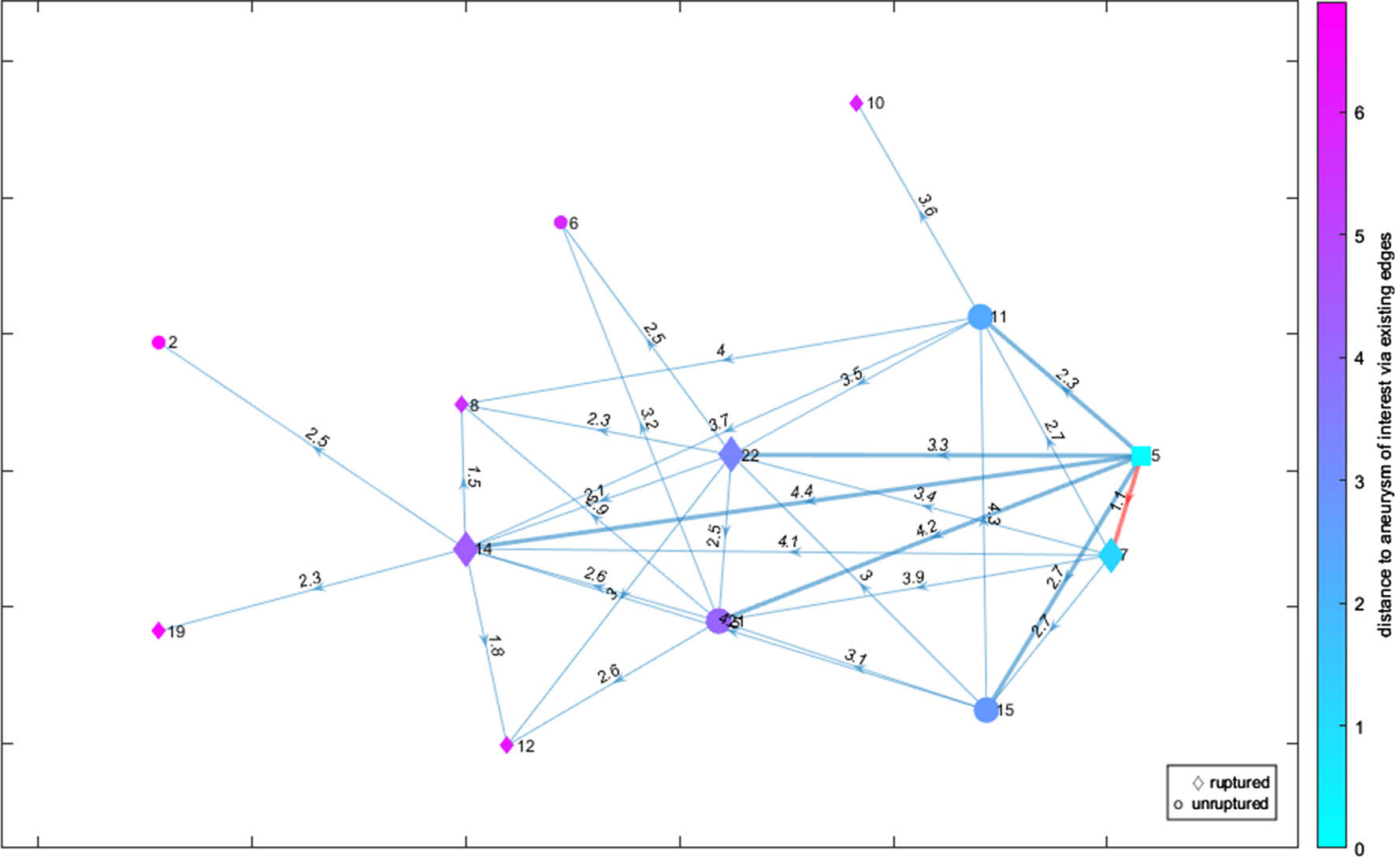
A directed force graph layout representing the *k* = 6 nearest neighbors (enhanced edges) including the highlighting of the most similar aneurysm (red edge) and a color-coding of the distance based on the color assigned to circular or diamond shapes representing the rupture state. The AOI is depicted as cyan-colored square

The last visualization is a directed graph (D), see Figs. 5 and 6. Each graph node represents an IA, and the directed edges indicate its *k* nearest neighbors. Each edge is labeled with the Euclidean distance between the two IAs/nodes that it connects. The glyph of each node shows the IA’s rupture status, with ruptured IAs being shown as a diamond shape, unruptured IAs as a circle and the AOI as a square. Hence, the illustration provided in Fig. 1 is reflected in this graph layout. The nodes are colored according to their distance to the AOI over the existing edges. The same colormap as for the heatmap was chosen. The AOI’s *k* nearest neighbors and their connecting edges are highlighted, with the most similar aneurysm’s connecting edge additionally being highlighted in a different color to grab the user’s attention.

The user can switch between two graph layouts: circle and force layout. The circle layout puts the AOI in the center and all other IAs on a set radius around it, regardless of them being nearest neighbors or not (see Fig. 5). The force layout arranges the nodes according to attracting and repelling forces (see Fig. 6). It matches the length of the edges to their weight, i.e., an edge’s length matches to the distance between the two nodes that it connects.

**Fig. 7:**
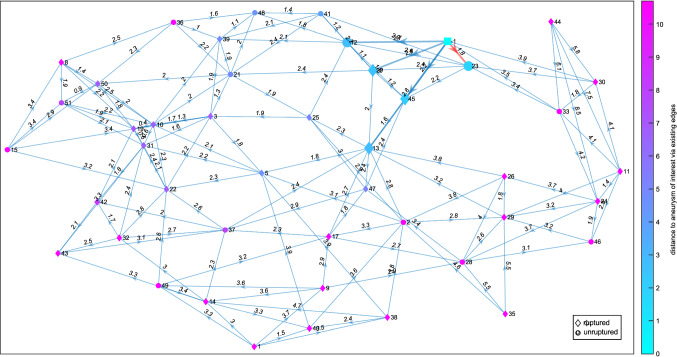
A full force graph representing the $k = 5$ nearest neighbors (enhanced edges) including the highlighting of the most similar aneurysm (red edge) and a color-coding of the distance based on the color assigned to circular or diamond shapes representing the rupture state. The AOI is depicted as cyan-colored square

**Fig. 8:**
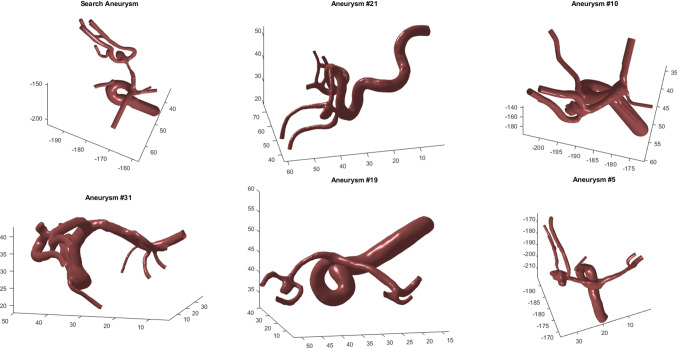
The user can select individual cases and depict the corresponding surface models, as well as an overview of the *k* most similar cases. A grid layout is used to represent the results. The user can rotate and zoom in for each individual model

The user can also decide whether the graph should feature all IAs in the database by selecting the “full” option (see Fig. 7), or only showing a “partial” graph (see Figs. 5 and 6), meaning only the AOI, its *k* nearest neighbors and those aneurysms’ *k* nearest neighbors. While the “full” layout gives a better overall view of how the AOI relates to the whole database, the graph quickly becomes crowded. Additionally, the amount of edges might occlude the highlighted edges of the AOI, especially for higher *k*. The “partial” option only shows the *k* closest IAs and their most similar, the former of which are the only ones relevant for the classification and thus give the user the necessary information and allow for easier interpretation.

Finally, the summary panel (panel (B) in Fig. 2) lists the results of the *k*-NN-based classifiers as plain text, including the majority used for *k*-NN, the weighted distances, the similarities, and how the results can be interpreted. The user is also notified whether there is the same amount of ruptured and unruptured IAs in the *k* neighborhood or if the distances calculated between ruptured and unruptured classes are smaller than a user-defined threshold (default value is 0.1). In these cases, the user is asked to increase *k*.

In order to support the medical users, a 3D view of the IA’s morphology is provided on demand. The user can get a 3D view of a single selected case as well as a grid layout-based depiction of the *k* most similar cases, see Fig. 8. The view can be updated via the settings panel (A).

In summary, the application allows the user to carry out CBR with an interactive *k*-NN search with outlier detection and feature selection. Outlier detection and feature selection are based on the analysis of the heatmap (C), graph layout (D) or scatterplot matrix (E). Via the “Define Selection” button in the settings panel (A), the user can select the IAs and/or features that should be used for the calculations. The heatmap and scatterplot matrix draw attention to the outliers and which features have a low contribution toward the classification or a high correlation with another more relevant feature. Thus, they guide the user when deciding which features might not give valuable input toward classification and give hints to which deselections might improve the results. The graphical presentation provides visual clues about similarity. As it is shown in Fig. 2, the majority of the five most similar cases to the AOI are ruptured, but the unruptured cases exhibit smaller distances (see the graph layout as well as the classification results in the summary panel). Thus, the AOI might have be a reduced risk of rupture and the physician can include this information into treatment planning. Finally, the user can use the patient IDs of the most similar cases (including classification of the AOI’s rupture risk) to look up the previous treatments in order to plan the best treatment for the AOI.

## Evaluation

We conducted an evaluation via a questionnaire with six physicians (two senior neurosurgeons with more than 15 years of experience, two advanced neurosurgeons with more than 2 years of experience and two novice medical doctors very familiar with intracranial aneurysms). In the questionnaire, the physicians were asked to rate each panel from useful to not useful with a 5-point Likert scale ranging from —(i.e., not helpful at all) to ++ (i.e., very helpful). For each component, they could add comments. Additionally, they were asked which component they found the most helpful, and which configuration of the directed graph they preferred.

**Fig. 9:**
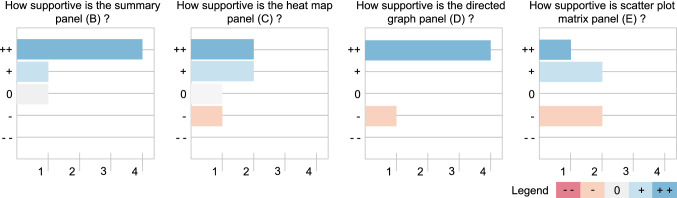
Overview of evaluation results regarding panels B, C, D and E (only one answer was possible using the Likert scale) is shown

**Fig. 10:**
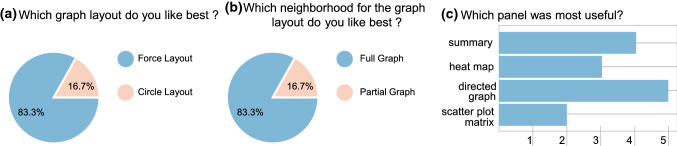
User preferences regarding the graph layout **a**, neighborhood size for graphs **b** and the overall preferred technique **c** are shown. For **c**, multiple answers were possible

Out of the six participants, four found the summary panel (B) very helpful, one found it somewhat helpful, and one neither helpful nor unhelpful, see Fig. 9. Additional comments varied, with one describing it as very useful and clear, while another found it confusing and would have wished for an aiding visualization and final summary of the classification results.

The heatmap panel (C) received more mixed feedback, with one rating it as somewhat unhelpful, one as neither helpful nor unhelpful, and two ratings for somewhat helpful and very helpful each. However, one physician commented they did not understand the purpose of the heatmap, while another found it statistically valuable.

The directed graph panel (D) was rated as most supportive, with four physicians finding it very helpful and one somewhat unhelpful. One physician declined to rate the directed graph. Almost all of the physicians preferred the force layout and the full graph over the circle layout and partial fullness option, with only one preferring the latter, see Fig. 10. They found an added visualization of the aneurysm models helpful.

The scatterplot matrix panel (E) received the worst feedback compared to the other visualization techniques, with one rating it as very helpful and two rating it somewhat helpful and somewhat unhelpful each. Again, one physician declined to rate. From the added comments, it was clear that the scatterplot matrix needs extensive explanation to be understood.

Finally, the physicians were asked to rate which of the four components they liked best, see Fig. 10c. They were allowed to select multiple answers. With most of the physicians (83%), the directed graph panel (D) was rated the most useful. The summary panel (B) came second with 67%. 50% liked the heatmap panel (C), while only 33% liked the scatterplot matrix.

## Conclusion

We presented an application to support physicians in the evaluation of intracranial aneurysms and their treatment decisions. Our application applies case-based reasoning to intracranial aneurysm patients and includes a heatmap visualization of all cases, a scatterplot matrix depiction of all cases’ attributes and a graphical representation to highlight the similarity of an aneurysm of interest to its *k* most similar cases. For intracranial aneurysms, a prediction of rupture risk always has an uncertainty due to patient-specific attributes and pathologic irregularities. However, the application visualizes this uncertainty due to the presentation of the most similar cases (instead of a single prediction) as well as the three classification results. The corresponding 3D views are provided individually for single cases as well as for the aneurysm of interest and its *k* most similar cases A user evaluation reveals the benefits of our methods, where the graphical representation based on the directed graph was rated as most useful.

In future work, we want to integrate further parameters, with focus on hemodynamic properties like wall shear stresses or inflow characteristics. In addition, the number of datasets is still rather low w.r.t. rupture risk assessment and we hope to add more datasets in future which is also motivated by the positive experiences of our evaluation partners. Our approach is not limited to intracranial aneurysms and can be easily adapted to other areas like epidemiological cohort study data.
